# Effectiveness of a Voice Training Program for Religious Officials in Turkey

**DOI:** 10.1007/s10943-024-02052-1

**Published:** 2024-04-25

**Authors:** Rıza Korhan Sezin, Özlem Yaşar, İbrahim Erensoy

**Affiliations:** Department of Speech and Language Therapy, Faculty of Health Sciences, Ondokuz Mayıs University, Kurupelit-Samsun, Turkey

**Keywords:** Acoustic measurements, Islamic religious officials, Vocal hygiene, Voice Handicap Index, Voice training

## Abstract

This study examines the effectiveness of a voice training program designed for Islamic religious officials, who are occupational voice users with a significant vocal load. The participants included 34 healthy religious officials whose acoustic measures were within normal voice ranges for healthy adults (jitter < 1%; shimmer < 3%) and reported no voice complaints. Participants were randomly divided into two groups (experimental, *n* = 17; control, *n* = 17). The two-stage voice training program consisted of 32 sessions over 8 weeks with informative and voice exercise stages. Objective and subjective voice measurements were performed at the beginning and end of the research. Objective measurements included fundamental frequency, percentage of vocal pitch perturbation (jitter), percentage of vocal intensity perturbation (shimmer), and harmonics-to-noise ratio. Subjective voice measurements included the Singing Voice Handicap Index, Vocal Fatigue Index (VFI), and Voice-Related Quality of Life (V-RQoL) scores. All initial measurements other than VFI scores were within acceptable limits for both groups. There were no significant differences between the groups initially (*p* > 0.05) and no significant changes in the control group in the second evaluation (*p* > 0.05). However, there was significant improvement in the experimental group after the training program in all measures, including VFI scores (*p* < 0.05). This study shows the positive results of a voice training program. Voice training should be integrated into the formal education of occupational voice users or in-service training programs of relevant institutions.

## Introduction

Occupational voice users, who constitute up to one-third of all individuals in the workforce, must meet certain vocal requirements to perform their jobs and financially support themselves (Buckley & Carey, 2022; Pomaville et al., 2020; Vilkman, 2000). Among these professionals, the job requirements of Islamic religious officials are unique. Although limited research has been conducted to date on the impacts of the vocal workload and vocal demands of this population, their work in mosques involves a heavy vocal load, including the recitation of Islamic memorial services and daily prayers. Similar to occupational voice users who sing or act, Islamic religious officials use their voices for long periods at high volumes and with varying pitches (Büyükatalay et al., 2020; Sarıca, 2018). Furthermore, this vocal load largely entails the recitation of holy texts in Arabic, which requires many religious officials to use vocal behaviors that differ from those of their native language, as is the case in Turkey. It is also important to note that, in Turkey, these officials do not undergo formal vocal health education or training (Büyükatalay et al., 2020; Fritzel, 1996; Subasi et al., 2023; Verdolini & Ramig, 2001). Given these circumstances, without vocal health protection or a knowledge of functional vocal techniques, these professionals are prone to vocal misuse and voice disorders may thus be inevitable. It is important that voice training be provided for the benefit of these professionals. Speech and language therapists advocate such interventions for occupational voice users in various fields to prevent serious voice disorders (Hazlett et al., 2011; Sezin et al., 2020).

The literature contains examples of studies evaluating voice training interventions with positive outcomes regarding the vocal health and vocal techniques of occupational voice users. For example, Pomaville et al. (2020) evaluated the effectiveness of vocal hygiene education for vocal performers. They concluded that increased knowledge of the larynx, voice production, and vocal hygiene led to improved vocal care and a decrease in symptoms such as throat irritation and vocal fatigue. Richter et al. (2016) conducted a study to evaluate the effectiveness of voice training for student teachers. Their training program was integrated into a teaching education program for a duration of 1.5 years and consisted of ten 90-min sessions in total. Open-access counseling was also provided, which allowed participants the opportunity to contact the researchers and ask questions regarding daily hygiene habits. Richter et al. (2016) stated that their findings clearly revealed the positive impact of voice training. However, they also stressed the importance of the quality and duration of interventions to be applied, citing negative examples from the literature.

In contrast, some studies have demonstrated no clear evidence in favor of voice training interventions. For example, Timmermans et al. (2011) examined the effectiveness of a short voice training program for future teachers and observed no significant difference between the experimental group and the control group. A similar outcome was reported by Duffy and Hazlett (2004), who evaluated the efficiency of voice programs designed for student teachers and reported that the assessment tools revealed no differences between the experimental and control groups. In both of these studies, the researchers theorized that the training period was not long enough for significant improvement to occur. Although many studies have been conducted in this field, the literature reflects a high degree of diversity in terms of contents, duration, and amount of sessions of the conducted interventions (López et al., 2017; Vermeulen et al., 2022). Furthermore, it has been noted that some of these studies have important limitations such as the lack of a control group or nonrandomized designs. The need for further research in this field has been stressed in the majority of the studies conducted to date (Nusseck et al., 2021; Richter et al., 2016).

In light of the acknowledged gaps in the literature, the present study was undertaken with the aim of examining the effectiveness of a voice training program designed for Islamic religious officials based on specific objective and perceptual parameters.

## Methodology

### Ethical Considerations

Ethics committee approval was obtained on June 30, 2022, from Ondokuz Mayıs University (Samsun, Turkey) with protocol number 2022-576. Written informed consent was obtained from all participants before the research began.

### Participants

Communication was established with volunteers from the local office of the Directorate of Religious Affairs, where initial interviews were held. Volunteers who presented with voice disorder complaints were directed to an ear, nose, and throat clinic following those initial interviews. Volunteers who did not report any voice-related complaints were randomly divided into the experimental and control groups. The randomization process was carried out using the computer-based Research Randomizer program available at randomizer.org. All volunteers were informed about the randomization process, and the participants in the control group were given the opportunity to benefit from the same training program after the research was concluded. After these steps, participants underwent objective and subjective voice evaluations.

The power analysis tools of IBM SPSS Statistics for Windows, version 22 (IBM Corp., Armonk, NY, USA), were used to assess the sample size of the study. The groups were planned with 20 participants, although the number decreased to 17 in each group due to dropouts.

The participants were between 29 and 61 years of age and had no history of voice, systemic, or neurological disorders. None of the participants had voice complaints at the beginning of or during the study. All participants were men, as mostly men serve as Islamic religious officials (Büyükatalay et al., 2020). Demographic information was collected using a participant information form designed by the authors of the study. The inclusion and exclusion criteria are listed in Table 1.

**Table 1:** Inclusion and exclusion criteria for participation

Inclusion criteria	Exclusion criteria
Being a full-time religious official currently employed	Not being a full-time religious official currently employed
No complaints of voice disorder	Voice disorder complaints
No upper respiratory tract infection in the last month	Upper respiratory tract infection in the last month
No use of medication affecting the voice on the evaluation days	Use of medication affecting the voice on evaluation days
No systemic or neurological disease	Systemic or neurological disease
Jitter percentage value below 1%	Jitter percentage value above 1%
Shimmer percentage value below 3%	Shimmer percentage value above 3%

The study enrolled 34 participants, with these religious officials divided into two groups. In the control group, ages ranged from 29 to 61 (mean: 44.35 ± 9.8) years, while the age range of the experimental group was 30–60 (mean: 43.7 ± 8.3) years. All participants were male, and none reported any kind of reflux, allergies, or smoking. In the experimental group, participants had 11–38 (mean: 23.7 ± 7.9) years of professional experience as Islamic religious officials, while the control group had 10–36 (mean: 23.23 ± 8.04) years of experience in this field. The participants’ demographic characteristics are presented in Table 2.

**Table 2:** Participants’ demographic characteristics (all participants were male)

Variables	Experimental group (*n* = 17), mean ± SD	Control group (*n* = 17), mean ± SD	*p*
Age, years, mean ± SD	43.76 ± 8.38	44.35 ± 9.82	0.852*
Water consumption, liters, mean ± SD	2.38 ± 0.32	2.32 ± 0.29	0.658**
Duration of professional experience, years, mean ± SD	23.70 ± 7.94	23.23 ± 8.04	0.923*
*Marital status, n (%)*			
Married	14 (82.4)	15 (88.2)	1.000^+^
Single	3 (17.6)	2 (11.8)	
Number of children, mean ± SD	2.11 ± 1.21	2.23 ± 1.14	0.892*

*Independent-samples *t* test, **Mann–Whitney *U* test, ^+^Fisher’s exact test

## Measures

### Objective Voice Evaluations

The Praat program was used for objective evaluations (Boersma & Weenink, 2002). As computer-based software, Praat facilitates the acoustic analysis of sound samples and provides objective data. The low cost and ease of use of this tool make it highly preferred for the observation of laryngeal status (Boersma & Weenink, 2002; Richardson et al., 2019). The parameters evaluated with the Praat program were fundamental frequency (F0), percentage of vocal pitch perturbation (jitter), percentage of vocal intensity perturbation (shimmer), and the harmonics-to-noise ratio (HNR). Participants were asked to phonate and sustain the /a/ vowel in a modal register with normal pitch and loudness in a sitting position 3 times repeatedly for 8 s in their “most comfortable tone”, and the mean scores were recorded. The middle 3-s portion of each sample was analyzed. Recordings were made in the Ondokuz Mayıs University Speech and Language Disorders Education, Research, and Application Center (OMÜKOM) with a laptop computer running the Windows operating system in a soundproof room in which the ambient noise level was below 50 dB SPL. The noise level of the room was measured using the Decibel X: Sound Level Meter application (SkyPaw Co., Ltd., Hanoi, Vietnam). A Shure PGA58 cardioid microphone (Shure Inc., Niles, IL, USA) was used for the recordings together with a Creative Labs Sound Blaster sound card (Creative Technology, Ltd., Singapore). The microphone was positioned at a 90° angle with a microphone-to-mouth distance of 10 cm following the recommendations of Titze and Winholtz (1993). All audio recordings were performed with a sample rate of 44.1 kHz and 16-bit resolution. The obtained sound recordings were saved in the WAV format and transferred to the Praat program. Objective voice analysis was performed for all participants before and after the voice training process.

### Subjective Voice Evaluations

For subjective voice evaluations, three self-administered questionnaires were used. These included the Turkish versions of the Singing Voice Handicap Index (SVHI) (Kiliç et al., 2008), Vocal Fatigue Index (VFI) (Sirin et al., 2020), and Voice-Related Quality of Life Scale (V-RQoL) (Tezcaner & Aksoy, 2017). All three subjective voice evaluation tools were administered to all participants before and after the voice training process.

The SVHI is a perceptual assessment tool with which individuals evaluate their own singing voices. It was originally developed by Cohen et al. (2007). There are two versions of the SVHI; the original version consists of 36 questions and a second shortened version, which was used in the present study, consists of 10 questions (Cohen et al., 2007, 2008; Sobol et al., 2020).

The VFI is also a self-report tool. Vocal fatigue is a term describing a multifactorial phenomenon that essentially involves a set of self-perceived symptoms of prolonged vocal work. These symptoms include excessive vocal effort, muscle tension, a feeling of discomfort or pain in the larynx, and a decrease in vocal efficiency. The VFI questionnaire aims to determine the presence of vocal fatigue and measure its symptoms. It is recognized as a reliable tool for identifying individuals who suffer from this phenomenon (Anand et al., 2021; Nanjundeswaran et al., 2015).

The last subjective assessment tool used in this study was the V-RQoL. Quality of life is one of the basic indicators of an individual’s state of health and well-being according to the World Health Organization (Hogikyan & Sethuraman, 1999). Comprising two subscales of social-emotional and physical functioning domains, each with 10 items, this questionnaire has been proven to be valid and reliable in addressing the quality of life of individuals in the context of voice (Gasparini & Behlau, 2009; Nusseck et al., 2020).

### Voice Training Program

Before the planning of the holistic voice training program, the corresponding author held a meeting with five experienced speech and language therapists and two religious officials to discuss the contents of the program. Building on those expert opinions, the voice training program was formalized after reviewing the relevant literature (Behrman & Haskell, 2019; Verdolini Abbott, 2008). It consisted of 32 sessions for 8 weeks and included both informative and voice exercise stages. In the informative stage, which comprised 8 sessions, basic information about vocal mechanisms, hygiene, anatomy, and the physiology of voice production was provided through presentations that involved visual materials and examples. In the voice exercise stage, which comprised 24 sessions, demonstrations and supervised applications of posture, breathing, vocal warm-ups, resonance, and projection exercises took place. In both stages, extra time was allotted for interaction, consultation, and questions and answers.

### Statistical Analysis

Data analysis was performed using IBM SPSS Statistics for Windows, Version 22 (IBM Corp.). Descriptive statistics were given as mean ± standard deviation for normally distributed variables and as median values for non-normally distributed variables. An independent t test was used to determine the significance of the differences in variables between the groups for normally distributed values, while the Mann–Whitney *U* test was used as a nonparametric test to evaluate differences between the groups when assumptions of the normality and homogeneity of variance were violated. To compare the groups before and after the voice training program, the paired-samples t test and Wilcoxon test were used. Mean total scores were used for the analysis of the self-report questionnaires. Values of *p* < 0.05 were accepted as statistically significant.

## Results

Variables were analyzed to identify differences between the experimental and control groups before and after the voice training program was administered to the experimental group. In Table 3, a comparison is presented of the acoustic variables and patient-reported outcome measures between groups before the voice training program. No statistical significance was found in the acoustic parameters and patient-reported outcome measure scores before the voice training program.

**Table 3:** Distribution of variables in the experimental and control groups before the voice training program

Variables	Experimental group (*n* = 17), mean ± SD	Control group (*n* = 17), mean ± SD	*p*
Maximum phonation time, seconds	19.58 ± 3.42	19.64 ± 2.91	0.957*
SVHI score	18.00 ± 6.28	16.52 ± 5.91	0.487*
V-RQoL score	68.00 ± 22.65	67.79 ± 15.65	0.976*
VFI score	29.05 ± 12.93	28.05 ± 12.16	0.818*
F0, Hz	126.52 ± 12.70	123.70 ± 12.73	0.522*
Jitter, %	0.27 ± 0.11	0.28 ± 0.09	0.669*
Shimmer, %	1.76 ± 0.51	1.75 ± 0.46	0.943*
HNR	25.44 ± 2.71	25.10 ± 2.31	0.692*

*SVHI* Singing Voice Handicap Index, *V-RQoL* Voice-Related Quality of Life Scale, *VFI* Vocal Fatigue Index, *F0* fundamental frequency, Jitter percentage of vocal pitch perturbation, Shimmer percentage of vocal intensity perturbation, *HNR* harmonics-to-noise ratio

*Independent-samples *t* test

In Table 4, comparisons of acoustic variables and self-reported outcome measure scores between the groups after the voice training program are presented. All parameters except *F*0 (*p* = 0.594) were found to be statistically significantly different between the groups (*p* < 0.05).

**Table 4:** Distribution of variables in the experimental and control groups after the voice training program

Variables	Experimental group (*n* = 17), mean ± SD	Control group (*n* = 17), mean ± SD	
Maximum phonation time, seconds	22.64 ± 2.34	19.58 ± 2.18	0.001**
SVHI score	12.17 ± 8.67	17.35 ± 5.92	0.013**
V-RQoL score	86.76 ± 15.75	69.41 ± 12.88	< 0.001**
VFI score	19.82 ± 8.97	31.76 ± 11.82	0.002*
F0, Hz	124.88 ± 10.30	123.00 ± 10.05	0.594*
Jitter, %	0.21 ± 0.04	0.28 ± 0.09	0.018**
Shimmer, %	1.41 ± 0.34	1.79 ± 0.55	0.020**
HNR	26.76 ± 2.10	25.16 ± 2.15	0.037*

*SVHI* Singing Voice Handicap Index, *V-RQoL* Voice-Related Quality of Life Scale, *VFI* Vocal Fatigue Index, *F0* fundamental frequency, Jitter percentage of vocal pitch perturbation, Shimmer percentage of vocal intensity perturbation, *HNR* harmonics-to-noise ratio

*Independent-samples *t* test; **Mann–Whitney *U* test

As seen in Table 5, paired-sample *t* tests were conducted to compare the variables before and after the members of the experimental group completed the voice training program. Among the acoustic parameters, the shimmer value was decreased (1.41 ± 0.34) and the HNR value was increased (26.76 ± 2.1) after the training program. Participants reported less handicap for their singing voices as the mean SVHI score decreased (12.17 ± 8.67) after the training program. They also reported fewer complaints about vocal fatigue, reflected in the decreased mean VFI score (19.82 ± 8.97). No significant differences were found for the control group upon the completion of the study.

**Table 5:** Comparisons of variables before and after the voice training program

	Variables	Before (*n* = 17), mean ± SD	After (*n* = 17), mean ± SD	*p*
Experimental group	Maximum phonation time, seconds	19.58 ± 3.42	22.64 ± 2.34	< 0.001*
	SVHI score	18.00 ± 6.28	12.17 ± 8.67	0.006**
	V-RQoL score	68.00 ± 22.65	86.76 ± 15.75	0.002**
	VFI score	29.05 ± 12.93	19.82 ± 8.97	0.015*
	F0, Hz	126.52 ± 12.70	124.88 ± 10.30	0.330*
	Jitter, %	0.27 ± 0.11	0.21 ± 0.04	0.013**
	Shimmer, %	1.76 ± 0.51	1.41 ± 0.34	0.004**
	HNR	25.44 ± 2.71	26.76 ± 2.10	0.016*
Control group	Maximum phonation time, seconds	19.64 ± 2.91	19.58 ± 2.18	0.768**
	SVHI score	16.52 ± 5.91	17.35 ± 5.92	0.305*
	V-RQoL score	67.79 ± 15.65	69.41 ± 12.88	0.387*
	VFI score	28.05 ± 12.16	31.76 ± 11.82	0.009*
	F0, Hz	123.70 ± 12.73	123.00 ± 10.05	0.562*
	Jitter, %	0.28 ± 0.09	0.28 ± 0.09	0.407**
	Shimmer, %	1.75 ± 0.46	1.79 ± 0.55	0.493*
	HNR	25.10 ± 2.31	25.16 ± 2.15	0.708*

*SVHI* Singing Voice Handicap Index, *V-RQoL* Voice-Related Quality of Life Scale, *VFI* Vocal Fatigue Index, *F0* fundamental frequency, Jitter percentage of vocal pitch perturbation, Shimmer percentage of vocal intensity perturbation, *HNR* harmonics-to-noise ratio

*Paired-samples *t* test; **Wilcoxon signed-ranks test

## Discussion

Islamic religious officials are faced with significant vocal loads in the course of their work as their daily responsibilities include reciting memorial services and prayers in Arabic (Büyükatalay et al., 2020; Subasi et al., 2023). Thus, as occupational voice users, they are prone to vocal misuse. However, limited research to date has addressed the vocal workload and vocal demands of this population. The present study aimed to examine the effectiveness of a voice training program designed for Islamic religious officials on specific acoustic parameters and self-reported outcome measures.

No significant differences were observed between the control group and the experimental group in the considered acoustic parameters or self-administered questionnaires before the voice training program was conducted. However, after the experimental group completed the training program, significant differences emerged between the groups for all acoustic and self-report outcome measures except the *F*0 value. *F*0 did not vary significantly and was in the acceptable range for both groups (Richardson et al., 2019; Titze & Winholtz, 1993).

Interestingly, although the participants did not report having any voice disorders, the overall VFI scores of both groups were remarkably high before the training program. This finding may support the claim that Islamic religious officials are generally subjected to heavy vocal demands, leading them to report vocal fatigue. The post-test VFI scores of the experimental group showed significant improvement.

Although the differences did not reach the level of statistical significance, there were also slight improvements in some post-test values of the control group including shimmer and the self-reported SVHI score. The participants in the control group were not offered the same education and training until after the completion of the study, but they may have had personal interactions with participants from the experimental group, who may have shared information about the practices of the voice training program on their own. Even without such interactions, the slight improvements observed in the control group could have resulted from the purpose of the study having been explained to all participants. Participating in voice-focused research may have generated more awareness of vocal care in the control group as well as the experimental group, inspiring participants to contemplate their vocal behaviors more seriously even before receiving any training.

The results of this study showed significant improvement in the self-reported outcome measures of the experimental group after the training program was concluded. Religious officials in the experimental group reported less handicap for their voices as reflected by decreased SVHI scores after the voice training program. They also had fewer complaints about vocal fatigue according to the decrease in VFI scores. There are conflicting results in the literature regarding the analysis of self-reported perceptual measures. Some authors found that the results of perceptual analysis did not consistently correlate with quantitative acoustic findings (Cheng & Woo, 2010). This inconsistency is understandable as the impact of voice alterations may be perceived differently by different individuals (Karlsen et al., 2020). In some studies, it was concluded that even when the acoustic parameters improved significantly, the participants’ self-reported outcome measures did not improve accordingly (Chen et al., 2007; López et al., 2017; Niebudek-Bogusz et al., 2010). In the present study, in contrast, the post-training self-reports reflected higher levels of improvement compared to the acoustic parameters. This result may be attributed to increased awareness of voice-related issues among the participants. However, it may also reflect an unrealistic inflation of the self-report scores of some participants due to their satisfaction with the voice training program and an ensuing increase in their self-confidence levels.

As acoustic parameters, the jitter and shimmer values of the experimental group were found to have decreased significantly in the post-training evaluation, whereas the mean HNR value increased. As noted above, HNR is an acoustic analysis parameter reflecting the ratio between periodic and non-periodic components of speech sound signals (Murphy & Akande, 2005). Among these variables, the jitter and shimmer values were particularly highly improved in the experimental group. There are similar results in the literature, with previous studies showing improvements in acoustic parameters including jitter, shimmer, and HNR after the implementation of voice training. However, the methodologies of previous studies must be taken into consideration while evaluating their results. Various methodological differences exist among them despite similarities in some cases regarding the number, duration, or contents of the training program sessions. In one of these studies that involved training program contents similar to those applied in the present study, Nusseck et al. (2021) examined the outcomes of ten 90-min sessions consisting of lectures on vocal mechanisms and vocal hygiene as well as applications of vocal exercises. They stated that their findings confirmed the importance of voice training. In another study conducted by López et al. (2017), the training similarly consisted of lectures on voice production and vocal hygiene followed by applied vocal exercises. The authors reported statistically significant improvements in acoustic and subjective voice parameters.

### Strengths and Limitations

Our study has both strengths and limitations. The major strength of the study is that it raises awareness about voice use and vocal load among Islamic officials as an under-studied group of occupational voice users. This study has revealed the needs of that group and the positive outcomes of a voice training program designed for religious officials by speech and language pathologists.

This study also has three main limitations. The first is the absence of laryngeal imaging. As recommended by the European Laryngological Society and the American Speech-Language-Hearing Association, laryngeal examination via imaging devices such as laryngoscopy can provide valuable data both before and after the implementation of education and training programs (Lechien et al., 2023; Patel et al., 2018). In this study, self-reported complaints of voice disorders constituted an initial exclusion criterion for participation. However, in the preliminary assessments, participants whose results deviated from the expected ranges of acoustic measurements were also excluded from the study (Friedrich & Dejonckere, 2005; Patel et al., 2018), with the intention of offsetting the impact of the lack of more formal laryngeal examinations. The second limitation is the sample size, which was reduced over the course of the research because of dropouts, reducing the strength of the study and making it more difficult to generalize the outcomes. The specialized nature of the participants’ profession inherently limited the population of the research, which was conducted in a single center. In future studies, the training program developed here could be expanded to the religious professionals of multiple Turkish provinces to obtain comparative results with a larger sample. The third limitation is the absence of follow-up evaluations. After the conclusion of the research, some of the participants were transferred by the Directorate of Religious Affairs to work in other provinces, which made it difficult to contact them for follow-up evaluations involving acoustic measurements. However, the evaluation of the long-term effects of this education and training program could provide valuable guidance in the design of future training programs.

## Conclusion

Despite the limitations recognized above, this study has clearly demonstrated the beneficial outcomes of a voice education and training program designed for Islamic religious officials. In Turkey, these officials do not normally receive any formal voice education or training, but it is widely recommended that voice education and training be integrated into the formal education processes of occupational voice users (Meier & Beushausen, 2021; Rantala et al., 2002), a broad category of employment to which Islamic religious officials belong. An alternative option might entail integrating such education into the in-service training programs of related institutions.
